# Epstein-Barr Virus Load Correlates with Multiple Sclerosis-Associated Retrovirus Envelope Expression

**DOI:** 10.3390/biomedicines10020387

**Published:** 2022-02-05

**Authors:** Silvia Pérez-Pérez, María Inmaculada Domínguez-Mozo, María Ángel García-Martínez, Rubén Ballester-González, Israel Nieto-Gañán, Rafael Arroyo, Roberto Alvarez-Lafuente

**Affiliations:** 1 Environmental Factors in Degenerative Diseases Research Group, Hospital Clínico San Carlos, IdISSC, 28040 Madrid, Spain; silvip93@hotmail.com (S.P.-P.); mariadomomd@gmail.com (M.I.D.-M.); garcia.angel23@gmail.com (M.Á.G.-M.); 2 Immunology Department, Hospital Universitario Ramón y Cajal, 28034 Madrid, Spain; ruben.ballester@salud.madrid.org (R.B.-G.); israelganan@yahoo.es (I.N.-G.); 3 Neurology Department, Hospital Universitario Quironsalud Madrid, 28223 Madrid, Spain; rafaelarroyo09@gmail.com

**Keywords:** EBV, HERV-W, HHV-6A/B, multiple sclerosis, syncytin-1

## Abstract

pHERV-W ENV and syncytin-1, the envelope proteins of the human endogenous retrovirus W family (HERV-W), have been proposed as etiological factors for MS development. In addition, herpesviruses, such as the Epstein-Barr virus (EBV) and the human herpesvirus 6A/B (HHV-6A/B), have been also strongly associated with the disease. This work aims to study the possible link between viral loads and antibody titers against EBV and HHV-6A/B and the pHERV-W ENV/syncytin-1 protein/gene expression. For this purpose, we conducted a 12-month longitudinal study involving 98 RRMS patients. Peripheral blood samples were obtained from each patient. Serum antibody titers against EBV and HHV-6A/B were determined by ELISA, while viral loads were analyzed using qPCR. HLA MS-related alleles were also genotyped. pHERV-W ENV/syncytin-1 protein and gene expression levels in immune cells were assessed by flow cytometry and qPCR, respectively. We found that the 12-month variation of the pHERV-W ENV gene expression levels positively correlated with the variation of the EBV viral load, especially in those patients with high baseline EBV loads. Therefore, these results could support previous studies pointing to the transactivation of pHERV-W ENV by EBV. However, further studies are needed to better understand this possible relationship.

## 1. Introduction

Multiple sclerosis (MS) is a chronic, autoimmune, and demyelinating disease that affects the central nervous system (CNS). Although it has been known for more than a century, the actual causes of MS are not fully understood yet. While many theories have been proposed, the most widespread one points out that several environmental factors could trigger an autoimmune response in genetically susceptible individuals [1]. On the one hand, regarding genetics, certain HLA (human leukocyte antigen) alleles are considered the main genetic risk factors for MS [2], including—but not only limited to—DRB1*15:01 (the most closely associated one), DRB1*03:01, DRB1*13:03, DRB1*08:01, or DQB1*03:02 alleles [3]. On the other hand, viruses are one of the most studied environmental factors related to the disease. Herpesviruses such as the Epstein-Barr virus (EBV) or the human herpesvirus 6A/B have been proposed as triggers for MS development [4,5,6]. However, there are only theories regarding their involvement in the disease, such as the possible transactivation of retroviral elements [7,8].

Human endogenous retroviruses (HERV) are sequences derived from exogenous retroviruses that integrated their genomes thousands of years ago into human germ cell lines [9]. MSRV (multiple sclerosis-associated retrovirus) and ERVWE1 are the main members that constitute the HERV-W family, the most studied one regarding MS development [10]. MSRV, the founder member of the HERV-W family, can form complete viral particles, but its genomic location is unknown. In contrast, ERVWE1, located at 7q21.2, possesses some inactivating mutations that impede proviral formation. Several complete or partial HERV-W envelope sequences have been mapped throughout the human genome. Syncytin-1, the envelope protein of ERVWE1, is a fusogenic protein indispensable for the formation of the placental syncytiotrophoblast [11]. Since the MSRV location is unknown, the rest of the HERV-W envelope sequences have been named pHERV-W ENV. pHERV-W ENV and syncytin-1 share 94% sequence homology, hindering their discrimination at the protein level with current commercial antibodies [12]. These proteins have been proposed as etiological factors for MS development, due to their proinflammatory and neurotoxic properties [13].

This work aims to study the possible link between viral loads and antibody titers against EBV and HHV-6 and the pHERV-W ENV/syncytin-1 protein/gene expression, as well as the possible influence of demographic, clinical, radiological, and genetic factors on this relationship. We found that the 12-month variation of the *pHERV-W ENV* gene expression levels positively correlated with the variation of the EBV viral load, especially in those patients with high baseline EBV loads. Therefore, these results could support previous studies pointing to the transactivation of pHERV-W ENV by EBV.

## 2. Materials and Methods

### 2.1. Patients and Samples

We conducted a 12-month longitudinal study that involved 98 RRMS patients diagnosed at “Hospital Clínico San Carlos” (Madrid, Spain), according to the updated McDonald criteria [14]. The demographic and clinical features of these patients are shown in Table 1.

The study complied with the guidelines of the Declaration of Helsinki, and it was approved by our local ethics committee (Comité Ético de Investigación Clínica del Hospital Clínico San Carlos; C.P.-C.I. 16/070-E). Every patient involved in this study accepted their enrolment by signing an informed consent form.

Two peripheral blood samples were collected from every patient (baseline—0 M—and 12 months later—12 M). On the one hand, we collected one dry tube for serum obtention; this tube was centrifuged (900 g, 15 min), and samples were stored at −80 °C. On the other hand, we also collected another blood sample in a cell preparation tube (CPT^TM^, BD Vacutainer) for peripheral blood mononuclear cells (PBMC) isolation. CPT tubes were centrifuged (920 g, 30 min) and isolated PBMC were cryopreserved in FBS (fetal bovine serum) supplemented with DMSO 10% (dimethyl sulfoxide). Finally, they were stored in liquid nitrogen (−176 °C).

### 2.2. Variables of the Study

- Laboratory variables: titers of antibodies against EBV (anti-VCA (EBV viral capsid antigen), anti-EBNA-1 (Epstein-Barr nuclear antigen) IgG antibodies), and HHV-6A/B (IgG and IgM antibodies); pHERV-W ENV/syncytin-1 protein expression; *pHERV-W ENV* and *syncytin-1* gene expression; EBV and HHV-6A/B viral loads; MS-related class II HLA alleles genotype.
- Demographic variables: sex and age.
- Clinical variables: age of onset of MS, disease duration, treatment, disability (according to EDSS—expanded disability status scale—and MSSS—multiple sclerosis severity score), presence/absence of relapses, gadolinium-enhanced magnetic resonance imaging (MRI) lesions presence/absence, and progression of the disease after 12 months (defined depending on the baseline EDSS score: increase ≥1.5 points if baseline EDSS was 0; increase ≥1 point if baseline EDSS was ≥1 and ≤5; increase ≥0.5 points if baseline EDSS was ≥5.5).

### 2.3. Enzyme-Linked Immunosorbent Assay (ELISA)

The following commercial kits were used for the detection of antiviral antibodies: Captia^TM^ kits (Trinity Biotech, Wicklow, Ireland) for anti-VCA and anti-EBNA-1 IgG determination and Viditest kits (Vidia, Ltd.; Vestec, Czech Republic) for anti-HHV-6A/B IgG and IgM measurement.

### 2.4. Monoclonal Antibody against pHERV-W ENV/syncytin-1 Obtention

For pHERV-W ENV/syncytin-1 protein expression analysis, we synthesized a monoclonal antibody in collaboration with the Monoclonal Antibodies Unit of CNIO (National Cancer Research Center). The antibody production, using hybridoma technology, was conducted following their standardized and validated protocols [15]. Supplementary Figure S1 shows the immunocytochemistry, immunohistochemistry, and flow cytometry images obtained during the validation of the manufactured monoclonal antibody (hybridoma: CALI222B). The antibody validation file is also available online [16].

### 2.5. Flow Cytometry

For flow cytometry experiments, 500,000 live PBMC were used to determine pHERV-W ENV/syncytin-1 protein expression through an indirect staining method. First of all, Fc receptors were blocked using 2.5 μL of Human TruStain FcX (BioLegend Cat# 422302, RRID:AB_2818986) (10 min, RT). Afterwards, we added 500 ng of the primary monoclonal antibody (30 min, 4 °C). After washing, 1 μL of a PE-labelled goat secondary antibody against rat IgG was added (20 min, 4 °C). Finally, PBMC were washed and stained with a set of monoclonal antibodies against the following surface markers: CD3-PerCP (BD Biosciences Cat# 345766, RRID:AB_2783791), CD14-FITC (BD Biosciences Cat# 345784, RRID:AB_2868810), CD16-PE-Cy7 (BD Biosciences Cat# 557744, RRID:AB_396850), CD19-APC (BD Biosciences Cat# 345791, RRID:AB_2868817), CD45-APC-H7 (BD Biosciences Cat# 641417, RRID:AB_2800453), and CD56-BV421^TM^ (BD Biosciences Cat# 562751, RRID:AB_273205).

Stained PBMC were analyzed in a CytoFlex flow cytometer and data analysis was performed using Kaluza Flow Cytometry Analysis Software (Gallios^TM^ Kaluza, RRID:SCR_016700), both supplied by Becton Dickinson (Franklin Lakes, NJ, USA). An average of 50,000 events per sample were analyzed. For gating, a selection of monocytes and lymphocytes was made to exclude debris and apoptotic/dead cells, followed by the selection of singlets and CD45+ (a marker of PBMC) cells.

pHERV-W ENV/syncytin-1 protein expression levels were analyzed in each of the following cell populations: monocytes, B lymphocytes, T lymphocytes, and NK cells.

### 2.6. Nucleic Acids Isolation

RNA was isolated from PBMC using the QIAamp RNA Blood Mini kit (Qiagen, Valencia, CA, USA). Afterwards, it was treated with the TURBO DNA-free™ kit (Invitrogen, Carlsbad, CA, USA), following, in both cases, the manufacturer’s instructions.

For DNA obtention from PBMC, the QIAamp DNA Blood Mini (Qiagen, Valencia, CA, USA) was used, following the manufacturer’s instructions.

### 2.7. qPCR for pHERV-W ENV and syncytin-1 Gene Expression Quantification

cDNA was synthesized from RNA using the Transcriptor First Strand cDNA Synthesis kit (Roche Diagnostics, S.L., Barcelona, Spain), where negative controls (without retrotranscriptase) were included. For *pHERV-W ENV* and *syncytin-1* gene expression quantification, we followed our previously published protocol [17].

### 2.8. qPCR for EBV and HHV-6A/B Viral Loads Determination

EBV and HHV-6 viral load quantification was conducted following the method previously published by our group [18].

### 2.9. MS-Related HLA Class-II Alleles Genotyping

Class II HLA alleles genotype was determined by rSSO-PCR (sequence-specific oligonucleotide reverse PCR). LABTypeTM One Lambda kits (Thermo Fisher Scientific, Waltham, MA, USA) were used. Finally, the detection was performed using a FLEXMAP 3DTM instrument (Luminex Corporation, Austin, TX, USA).

### 2.10. Statistics

Firstly, normality was evaluated for each variable, according to the Kolmogorov–Smirnov test. Continuous variables were expressed as mean ± SD (standard deviation) if they were parametric, or as median (25th, 75th percentile) if they were not, and categorical variables were expressed as percentages.

For antibody titers and viral loads, only positive values were used in the analyses, except for anti-HHV-6A/B IgM titers, due to low seroprevalence reasons.

To study the possible link between the variation of viral load/antibody titers against EBV and HHV-6A/B, and the variation of the pHERV-W ENV/syncytin-1 protein/gene expression, the differences in each variable throughout the study were assessed using: (1) the paired samples t-tests, for parametric variables; (2) the Wilcoxon signed-rank test, for non-parametric variables; or (3) McNemar’s test, for categorical variables. If any of those changes were significant, a new variable resulting from the difference between the two points was then calculated (for example, (EBV load (12 M)–EBV load (0 M)).

Finally, we evaluated the possible relationship between these new variables. The relationships with the variation of the pHERV-W ENV/syncytin-1 protein/gene expression levels were assessed using: (1) the Pearson correlation coefficient or the Spearman’s rank correlation coefficient (parametric or non-parametric variables, respectively), for viral loads/antibody titers; (2) Student’s t-test or the Mann–Whitney U test (parametric or non-parametric variables, respectively), for prevalence values.

To analyze the possible impact of demographic, clinical, radiological, and genetic features on these relationships, the aforementioned process was repeated after stratifying the cohort accordingly, as well as considering baseline serological and viral load values (0 M). For continuous variables, the median values were selected as the cut-offs, except for age (the cut-off was 45 years old [19], proposed as the possible immunosenescence onset for MS patients) and EDSS (the cut-off was 3, proposed as the onset of irreversible disability [20]).

Subjects with missing data were omitted from the corresponding analyses. *p*-values <0.05 were considered statistically significant. All analyses were performed using SPSS version 21.0 (IBM SPSS Statistics, RRID:SCR_019096) and graphs were made using Prism version 5.0 (GraphPad Prism, RRID:SCR_002798).

## 3. Results

Before analyzing the possible link between the variation of viral loads/antibody titers against EBV and HHV-6A/B, and the variation of the pHERV-W ENV/syncytin-1 protein/gene expression, we evaluated the variation of each variable from the 0 M point to the 12 M point independently. We found significant variations in the following continuous variables: (1) anti-HHV-6A/B IgM antibodies (AU, median (P25–P75): 0 M = 3.42 (2.82–5.54); 12 M = 4.54 (2.66–9.67)—*p* = 1.41 × 10^−8^, Wilcoxon signed-rank test); (2) EBV viral load (copies/μg DNA, median (P25–P75): 0 M = 146.15 (73.60–310.40); 12 M = 228.57 (123.20–523.20)—*p* = 0.001, Wilcoxon signed-rank test); (3) HHV-6A/B viral load (copies/μg DNA, median (P25–P75): 0 M = 29.23 (18.40–38.40); 12 M = 7.20 (3.20–10.39)—*p* = 0.005, Wilcoxon signed-rank test); (4) pHERV-W ENV/syncytin-1 protein expression on non-classical (Mo3) monocytes (%, mean ± SD: 0 M = 93.70 ± 4.55; 12 M = 94.63 ± 8.78—*p* = 0.039, paired samples *t*-tests) and B lymphocytes (%, median (P25–P75): 0 M = 10.00 (6.41–14.04); 12 M = 12.91 (8.01–21.96)—*p* = 2.15 × 10^−4^, Wilcoxon signed-rank test); and (5) *pHERV-W ENV* gene expression (median (P25–P75): 0 M = 1.22 (0.81–2.32); 12 M = 1.41 (0.97–2.84)—*p* = 0.001, Wilcoxon signed-rank test).

Regarding qualitative variations (serological and qPCR prevalences), only the anti-HHV-6A/B IgM seroprevalence changed significantly between the 0 M and the 12 M points (*n*: positive-positive: 76, positive-negative: 0, negative-positive: 10, negative-negative: 11; *p* = 0.002, McNemar’s test).

Regarding HLA genotyping, the percentages of carriage (at least in heterozygosis) of the main MS-related HLA alleles were the following: DRB1*15:01 (42%), DRB1*03:01 (25%), DRB1*13:03 (7%), DRB1*08:01 (6%), and DQB1*03:02 (22%).

**Figure 1:**
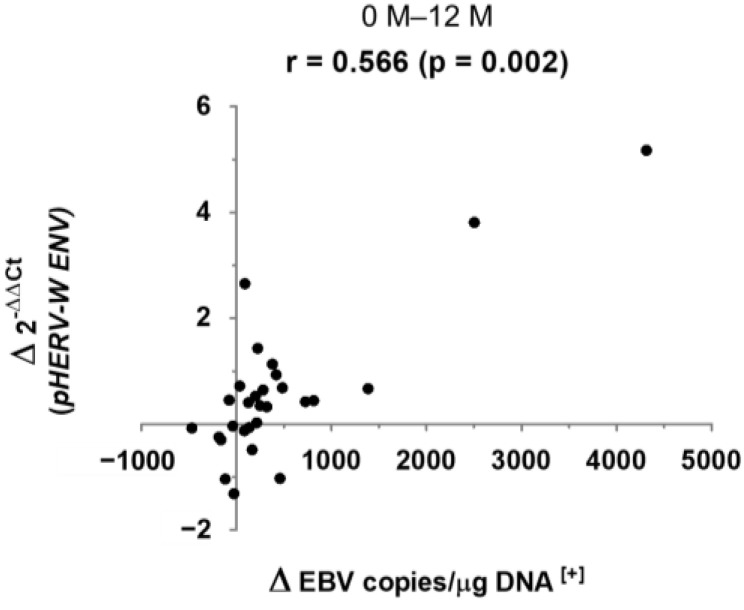
Correlation between the variation of EBV load and the variation of *pHERV-W ENV* gene expression, from 0 to 12 months, in RRMS patients. (r: Spearman’s correlation coefficient; Δ: variation; [+]: positive values).

**Figure 2:**
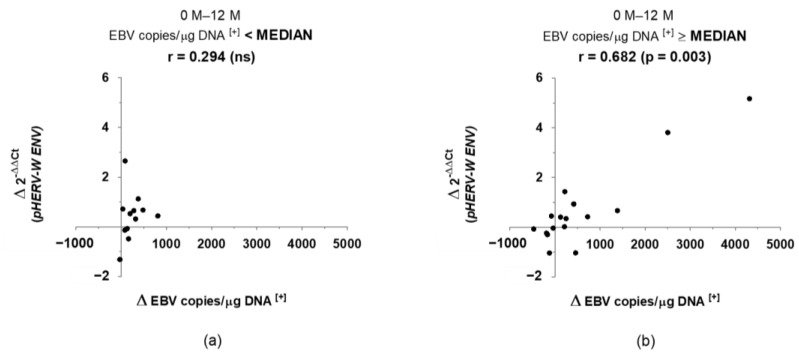
Correlation between the variation of EBV load and the variation of *pHERV-W ENV* gene expression, from 0 to 12 months, in RRMS patients, depending on the initial viral load: lower (**a**) or higher (**b**) than the median value (146 copies/μg DNA). (r: Spearman’s correlation coefficient; Δ: variation [+]: positive values).

After having identified the variables that changed significantly throughout the study, we proceeded to assess the possible correlations existing between them. We only found one significant correlation, with moderate-high strength, between the variation of EBV load and the variation of *pHERV-W ENV* gene expression, in a positive way (Figure 1). Moreover, taking into account the EBV baseline load values, this correlation was only found, with a stronger association, in those patients with an EBV baseline load higher than the total median value (146 copies/μg DNA), as shown in Figure 2.

**Figure 3:**
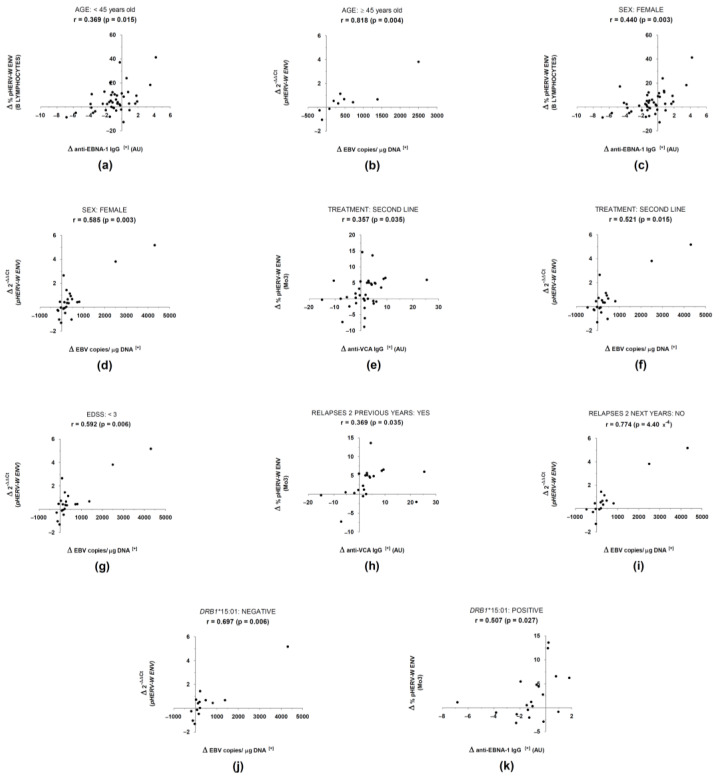
Influences of demographical (**a**–**d**), clinical (**e**–**i**), and genetic (**j**,**k**) factors on correlations between antiviral antibody titers and pHERV-W ENV/syncytin-1 expression. (AU: arbitrary units; r: Spearman’s Correlation Coefficient; Δ: variation; [+]: positive values).

Finally, regarding the possible influence of the aforementioned demographic, clinical, radiological, and genetic characteristics on the correlation between the variation of EBV load and the variation of *pHERV-W ENV* gene expression, correlations seemed to be more associated with the following factors (Figure 3): female, age ≥ 45 years old, second-line treatment (natalizumab), EDSS < 3, absence of relapses in the two years after 0 M sample collection and absence of the HLA DRB1*15:01 allele. On the contrary, we found no association after stratifying patients based on the age of onset of MS, disease duration, MSSS, gadolinium-enhanced MRI lesions, or progression of the disease.

## 4. Discussion

Although some pieces of the MS etiopathogenesis puzzle are still missing, there is no doubt of the joint involvement of genetic and environmental factors. The role of viruses—especially herpesviruses—in MS is almost undeniable, since many different researchers have come to this conclusion. For example, data reveal that EBV could trigger a strong humoral immune response in MS patients, with higher anti-EBNA-1 antibody titers compared to HD, which decrease after interferon therapy [21,22]. However, there is still a debate about the underlying mechanisms, with different theories being proposed. Regarding EBV, it has been proposed to overstimulate immune responses, as well as to be part of a possible cross-reaction with myelin proteins [23,24]. Other theories, however, propose a possible transactivation of HERV elements, including the HERV-W family envelope proteins [25]. This kind of process has already been reported, as in the case of the transactivation of HERV-K18 env by EBV in B lymphocytes [26].

After the discovery of MSRV at the end of the past century, a new framework for the study of MS emerged based on the possible involvement of this HERV—and its proteins—in both the development and the evolution of the disease [27,28]. Many researchers have proposed a possible link between pHERV-W ENV/syncytin-1 and MS [29]. Based on previously published studies, anti-pHERV-W ENV/syncytin-1 antibodies could be used as MS biomarkers, allowing discrimination from other diseases such as neuromyelitis optica [30,31]. However, this relationship is also surrounded by controversy, which justifies continuing delving into this issue.

In this work, we have found a significant, positive correlation between the 12-month variation of EBV load and the 12-month variation of *pHERV-W ENV* gene expression. Furthermore, this relationship seemed to be stronger in those patients with a high baseline EBV load (higher than the median value of all of the patients), with no significant association in patients with low EBV loads. The high strength of this result could suggest that the variation in EBV viral load is only effective upon *pHERV-W ENV* gene expression when EBV is highly present in an individual. Therefore, variations between low EBV loads would be irrelevant.

Previous works have already pointed out this relationship. For example, Mameli et al. found higher pHERV-W ENV expression levels in patients suffering from active mononucleosis (the most common disease caused by EBV), compared to HD [32]. However, there are also contrary voices and other studies have claimed that there is no relationship between EBV and pHERV-W ENV expression [33].

In spite of the aforementioned results, we have not found this correlation between pHERV-W ENV expression and anti-EBV antibodies (nor anti-VCA or anti-EBNA-1 IgG titers or prevalences). As has been published, serological titers could not be a faithful reflection of the viral situation [34,35], especially in patients with aberrant immune responses, such as the MS patients.

Regarding the possible influence of demographic, clinical, radiological, and genetic factors, the correlations were more associated with the following: females, age equal or higher than 45 years old, treatment with second-line therapy, EDSS lower than 3, absence of relapses in the two years after the start of the study, and absence of the MS-related class II HLA DRB1*15:01 allele. In general, these correlations followed the same trend previously found when analyzing the whole population. Moreover, correlations between the variation in EBV load and the variation in *pHERV-W ENV* were consistently the strongest (r ≥ 0.521), with some cases of a particularly highly strong association (r = 0.818). Thus, the results seemed to be robust enough to support our hypothesis of possible transactivation of *pHERV-W ENV* by EBV.

In relation to age, the finding of the strongest correlation between EBV and *pHERV-W ENV* in patients older than 45 years old could reflect an association between these factors and neurodegeneration, the effects of which are more evident in those patients. EBV has been associated with neurodegeneration in MS. This neurodegenerative process would be the target of temelimab—the anti-pHERV-W ENV monoclonal antibody in ongoing clinical trials, which has shown a potential anti-neurodegenerative effect in MS patients [36]. Therefore, the link between EBV and neurodegeneration, added to the possible anti-neurodegenerative effect of temelimab, makes our results robust and reliable.

As regards sex, an association with females could be in line with the presence of a *pHERV-W ENV* sequence (ERVWE2) in chromosome X—this sequence has been proposed to be the one involved in MS [37]. Another factor to take into consideration is the presence of relapses; the strong association in those patients who did not suffer relapses could be reflecting that the link between EBV and *pHERV-W ENV* is not involved in predicting the appearance of future relapses, and simply reflects the present situation.

In contrast to other previously published studies, we have not found any association in terms of disease progression (i.e., variation of EDSS). For example, in the studies published by Sotgiu et al. [38,39], they found that the presence of MSRV virions in the cerebrospinal fluid of MS patients at disease onset is associated with both a higher rate of relapses and the accumulation of disability. Unlike our study, which has a duration of 12 months, those studies were 3 and 6 years long, respectively; we believe that this is probably the reason why we have not been able to find an association with disease progression.

Regarding genetics, we have not found a correlation between the EBV load and the *pHERV-W ENV* gene expression in carriers of the DRB1:15*01 allele or any other of the MS-related alleles. While previous regression analyses conducted by other researchers [35] have shown the independent mechanisms of these MS risk factors (EBV and *DRB1*:15*01), we considered these results to be important and of interest for further analysis in future studies.

Concerning therapies, other groups have published that six-month treatment with interferon [40] and long-term treatment with natalizumab [41] can reduce the humoral immune response against pHERV-W ENV. While we found variations in several variables between the baseline and the 12-month samples, we found no association or effect of the different therapies included in this work (beta interferon/glatiramer acetate vs. natalizumab). However, these studies assessed anti-pHERV-W ENV antibody titers and did not directly assess pHERV-W ENV/syncytin-1 protein/gene expression.

The mechanism underlying the possible link found between EBV and pHERV-W ENV could be similar to the following. Immune cells infected by EBV would penetrate the CNS, where they would trigger an inflammatory environment. The release of proinflammatory molecules would lead to the overexpression of pHERV-W ENV sequences, which, in turn, would increase the release of proinflammatory factors, creating an endless loop and, in the end, causing neuroinflammation, demyelination, and tissular damage [7].

HERV expression has been reported to be strongly affected by many factors, both genetic and environmental [42,43]. The added value of the current work is to have conducted a longitudinal study that minimizes those differences, making it robust and reliable.

Among the work limitations, it has to be considered that the radiological data that we were able to collect from the clinical records were limited. This fact could be the reason for not having found an association with gadolinium-enhancing lesions, as we did in previous studies [44]. In addition, in spite of analyzing several variables and conducting the corresponding tests, we have not applied corrections for multiple comparisons due to the exploratory nature of this study.

Finally, regarding the latest study conducted by Bjornevick et al. [6], authors point to EBV as the leading cause for MS using VirScan, high-throughput technology for antiviral antibodies analysis. Although in this study HERV-W is not mentioned as a relevant factor for MS development, it is probably due to both technical limitations, in regards to post-translational modifications and the complexity of HERV sequences, and the possibility of not having taken into account endogenous retroviral sequences when preparing the library for the genomic analyses. Of course, it would be of great interest to be able to use VirScan for anti-pHERV-W ENV antibodies detection in further studies in order to get more evidence on the relationship between EBV and HERV-W in regards to MS. However, due to the particularities of HERV sequences, T-Scan—an-other high-throughput platform for identification of antigens recognized by T cells developed by the same research group [45], would be more suitable for HERV-related analysis.

In conclusion, the aforementioned results could support our hypothesis and the findings of previous studies suggesting a possible transactivation of *pHERV-W ENV* by EBV in peripheral blood mononuclear cells. It is important to note that this study is one of the most detailed ones so far aiming to investigate the relationship between herpesviruses and pHERV-W ENV, including a large cohort of RR-MS and a 12-month follow-up. However, more exhaustive and comprehensive studies will be needed to find out whether this is an MS-specific event and to establish whether this correlation is clinically relevant.

## Figures and Tables

**Table 1:** Demographic and clinical characteristics of patients included in this study at recruitment.

	RRMS
***n***	98
**Sex** (**% females**)	70.4
**Age** (**y.o., mean ± SD**)	40.2 ± 7.7
**Disease duration** (**m, median** (**P25–P75**))	118.0 (63.3–178.0)
**Treatment** (**%**)**:**	
**First line** (**beta interferon, glatiramer acetate**)	43.9
**Second line** (**natalizumab**)	56.1
**Treatment duration** (**m, median** (**P25–P75**))**:**	
**First line**	45.0 (24.0–62.0)
**Second line**	12.0 (2.0–27.0)
**EDSS** (**median** (**P25–P75**))	2.0 (1.0–3.0)
**MSSS** (**median** (**P25–P75**))	2.3 (0.8–4.2)
**Annualized relapse rate** (**median** (**P25–P75**))	0.6 (0.4–0.9)

y.o.: years old; m: months; P: percentile; SD: standard deviation.

## Data Availability

The data that support the findings of this study are available on request from the corresponding author. The data are not publicly available due to privacy or ethical restrictions.

## Supplementary Materials

The following supporting information can be downloaded at: https://www.mdpi.com/article/10.3390/biomedicines10020387/s1, Figure S1: immunocytochemistry (A), immunohistochemistry (B), and flow cytometry (C) experiments for the validation of the manufactured monoclonal antibody CALI222B.
