# Supplementary material for: Epstein-Barr Virus Load Correlates with Multiple Sclerosis-Associated Retrovirus Envelope Expression
Source: Biomedicines. 2022 Feb 5;10(2):387. doi: 10.3390/biomedicines10020387 (PMC8962350; doi:10.3390/biomedicines10020387)
Supplement: Supplementary file 1 [file biomedicines-10-00387-s001.zip › Supp_Fig1.pdf]

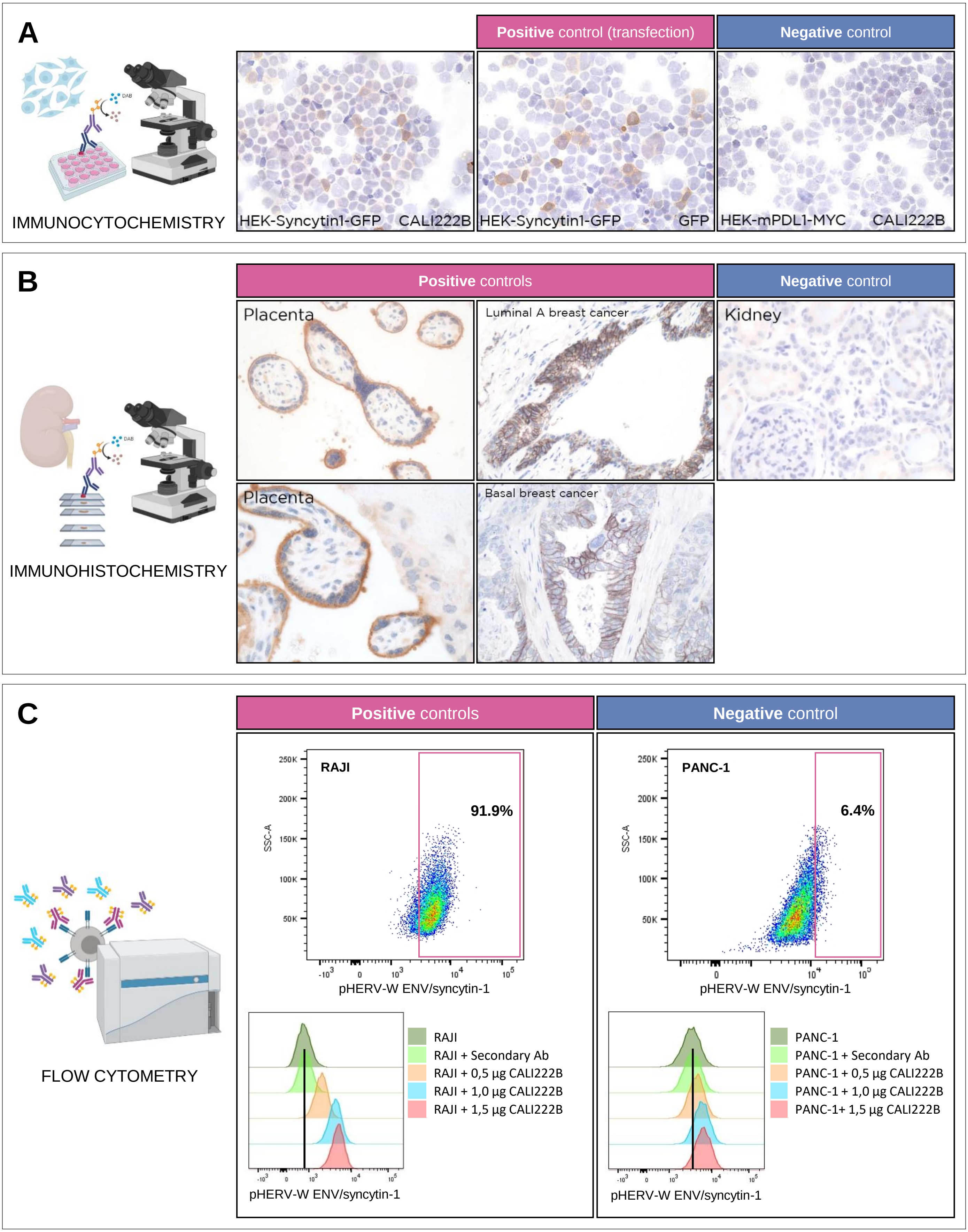


**Supplementary Figure S1.** Immunocytochemistry (A), immunohistochemistry (B) and flow cytometry (C) experiments for the validation of the manufactured monoclonal antibody CALI222B.

(GFP: green fluorescent protein; HEK: Human embryonic kidney; MYC: protooncogene; PANC-1: human pancreatic cancer cell line; PDL1: Programmed Death-ligand 1; RAJI: human lymphoid cell line)
